# Low Carbon sink capacity of Red Sea mangroves

**DOI:** 10.1038/s41598-017-10424-9

**Published:** 2017-08-29

**Authors:** Hanan Almahasheer, Oscar Serrano, Carlos M. Duarte, Ariane Arias-Ortiz, Pere Masque, Xabier Irigoien

**Affiliations:** 1 Biology Department, University of Dammam (UOD), Dammam, 31441-1982 Saudi Arabia; 2 School of Science, Centre for Marine Ecosystems Research, Edith Cowan University, 270 Joondalup Drive, Joondalup, Western Australia 6027 Australia; 3 King Abdullah University of Science and Technology (KAUST), Red Sea Research Center, Thuwal, 23955-6900 Saudi Arabia; 4 Universitat Autònoma de Barcelona, Departament de Física & Institut de Ciència i Tecnologia Ambientals, Barcelona, Spain; 5 The UWA Oceans Institute & School of Physics, University of Western Australia, 35 Stirling Highway, Crawley, 6009 Australia; 6 AZTI - Marine Research, Herrera Kaia, Portualdea z/g – 20110 Pasaia (Gipuzkoa), Pasaia, Spain; 7 IKERBASQUE, Basque Foundation for Science, Bilbao, Spain

## Abstract

Mangroves forests of A*vicennia marina* occupy about 135 km^2^ in the Red Sea and represent one of the most important vegetated communities in this otherwise arid and oligotrophic region. We assessed the soil organic carbon (C_org_) stocks, soil accretion rates (SAR; mm y^−1^) and soil C_org_ sequestration rates (g C_org_ m^−2^ yr^−1^) in 10 mangrove sites within four locations along the Saudi coast of the Central Red Sea. Soil C_org_ density and stock in Red Sea mangroves were among the lowest reported globally, with an average of 4 ± 0.3 mg C_org_ cm^−3^ and 43 ± 5 Mg C_org_ ha^−1^ (in 1 m-thick soils), respectively. Sequestration rates of C_org_, estimated at 3 ± 1 and 15 ± 1 g C_org_ m^−2^ yr^−1^ for the long (millennia) and short (last century) temporal scales, respectively, were also relatively low compared to mangrove habitats from more humid bioregions. In contrast, the accretion rates of Central Red Sea mangroves soils were within the range reported for global mangrove forests. The relatively low C_org_ sink capacity of Red Sea mangroves could be due to the extreme environmental conditions such as low rainfall, nutrient limitation and high temperature, reducing the growth rates of the mangroves and increasing soil respiration rates.

## Introduction

Mangrove forests supply important ecosystem services that support the livelihoods of coastal societies in the tropics and subtropics^1^. However, these extend beyond the local communities to yield, through their role as a globally-relevant carbon sinks, climate change mitigation benefits to the global community^2–5^. Although mangroves occupy only 0.5% of the area of the global coastal ocean^6^ and represent only 0.7% of the tropical forests of the world^7^, they account for about 1% and 14% of the carbon sequestered annually by the world’s forests and the global ocean, respectively^8^.

The intense organic carbon (C_org_) sequestration by mangrove forests is mainly due to their high net ecosystem production^9^, resulting in high loads of leaf litter and biomass to the soil, combined with their high capacity to trap and retain soil resulting in vertical accretion^10^ compared to other forests and coastal areas devoid of vegetation^11^. Further, high C_org_ preservation due to low oxygen availability^12, 13^ combined with lack of fires in the aquatic environment where mangroves thrive^3^, results in high carbon storage in mangroves^14, 15^.

Unfortunately, mangrove ecosystems have declined globally, with one third of the global mangrove area lost since World War II^16^. Mangrove deforestation leads to losses of their carbon sink capacity as well as greenhouse gas emissions from remineralization of the large C_org_ stocks that mangroves accrete^3, 14^. Annual CO_2_ emissions associated with mangrove deforestation have been estimated at 0.02 to 0.12 Pg C^14^, contributing to CO_2_ emissions through land-use change, the second largest anthropogenic source of CO_2_ to the atmosphere after fossil fuel combustion^17^.

The Red Sea has recently been identified as possibly the only mangrove province where mangroves are not only stable, but have expanded by 12% over the last four decades^18^. Lack of freshwater and soil inputs lead to acute nutrient limitation of Red Sea mangroves^19^, resulting in mangrove forests being dominated by dwarf trees^20^, similar to those in other arid areas (e.g. Gulf of California^21^ and areas lacking surface runoff such as the Indian River Lagoon in Florida^22^). Hence, we would expect mangrove forests in the Red Sea to have a relatively modest carbon sink capacity so that, despite the stability of the forests, their contribution to carbon sequestration would still be marginal. Yet, most estimates of carbon sequestration and stocks in mangrove forests have been, hitherto, derived from mangrove forests in the wet tropics^6, 8^, and whether those growing in the arid topics contribute significantly to carbon sequestration remains an open question. However, a recent assessment reports a C_org_ sequestration for dwarf mangrove forests in the arid shores of Baja California of, on average, 1000 Mg C_org_ ha^−1^ in 1.5 m-thick soils (adapted from ref. 23), similar to that found under some of the tallest tropical mangroves in the Mexican Pacific coast^23^, which has been linked to the stability of these forests. These observations question the assumption that dwarf mangroves in arid shores support low carbon sequestration rates and stocks.

Here we assess the C_org_ stock and sequestration rates and stocks supported by *Avicennia marina* mangrove forests in the Central Red Sea. We do so by combining measurements of soil C_org_ density down to 10 cm with soil chronologies derived from ^210^Pb and soil C_org_ density down to 1 m depth with soil chronologies derived from ^14^C, to estimate (a) the stock of C_org_ contained within the top meter of the soil, and (b) the burial rate of C_org_ over short-term (last 100 years) and long-term (last millennia) periods. Further, we use stable carbon and nitrogen isotopes of the organic matter in the soil and putative sources (mangroves, halophytes, seaweed, seagrass and seston) to estimate the potential contribution of different sources of C_org_ to mangrove soil carbon stocks.

## Results

The distribution of soil grain size differed among mangrove forests, with soils sampled at Thuwal Island, Economic City and Khor Alkharar having a sandy texture, (∼85% of sand fraction >0.063 mm) compared to loamy sand in Petro Rabigh (32% of mud <0.063 mm) (Table 1), where the clay and silt contents in the soils was 2 to 3-fold higher (*P* < 0.001), (Tukey HSD multiple comparison post-hoc test, *P* < 0.05). Mangrove forests at Thuwal Island grow in the coarsest soil (80% particles between 0.125 and 1 mm) compared to the other study sites (47 to 65% particles between 0.125 mm and 1 mm, Table 1).

**Table 1:** Mean (±SE) grain size fractions (%) and texture in mangrove soils from the four study sites in the Central Red Sea.

Location	*n*	% of Soil classification <1 mm					Texture
		Clay and Silt particles (<0.063 mm)	Very fine sand (<0.125 and >0.063 mm)	Fine sand (<0.25 and >0.125 mm)	Medium sand (<0.5 and >0.25 mm)	Coarse sand (<1 and >0.5 mm)	
Thuwal Island	65	10.2 ± 0.7^b^	11 ± 0.5^a^	25.5 ± 0.7^a^	29.8 ± 0.6^a^	23.5 ± 1^a^	Sand
Economic City	104	17.9 ± 0.7^b^	18.1 ± 0.6^a^	21.9 ± 0.4^a^	21.8 ± 0.5^a^	20.2 ± 0.7^a^	Sand
Petro Rabigh	78	31.6 ± 1.8^a^	21.1 ± 1.3^a^	23.1 ± 1.3^a^	13.7 ± 1^b^	10.5 ± 1.2^b^	Loamy sand
Khor Alkharar	65	16.2 ± 0.8^b^	19 ± 0.5^a^	25.6 ± 0.4^a^	21.9 ± 0.5^ab^	17.2 ± 0.8^ab^	Sand
**Averages**	312	19.4 ± 0.7	17.6 ± 0.5	23.7 ± 0.4	21.5 ± 0.5	17.8 ± 0.5	Sand
***R*** ^**2**^		0.7	0.8	0.6	0.9	0.9	
***F*** **ratio** (location)		11^**^	2.8 ^ns^	1.1 ^ns^	6.3^**^	3.9^*^	
***F*** **ratio** (depths nested-within-cores)		3.9^**^	5.6^**^	2.9^**^	6.4^**^	3.4^**^	

*R* ^2^ and *F* ratio correspond to the square-root results of the GLMMs testing the effect of independence of samples. Study site and soil depth (nested within cores) as fixed factors, whereas replicate cores within sites was treated as random factor. *P value between 0.01 and 0.05, **P value < 0.01 for significant differences whereas (^ns^) means not significant. Columns linked with the same letter did not differ significantly among themselves (Tukey HSD multiple comparison post-hoc test, *P* > 0.05).

**Figure 1:**
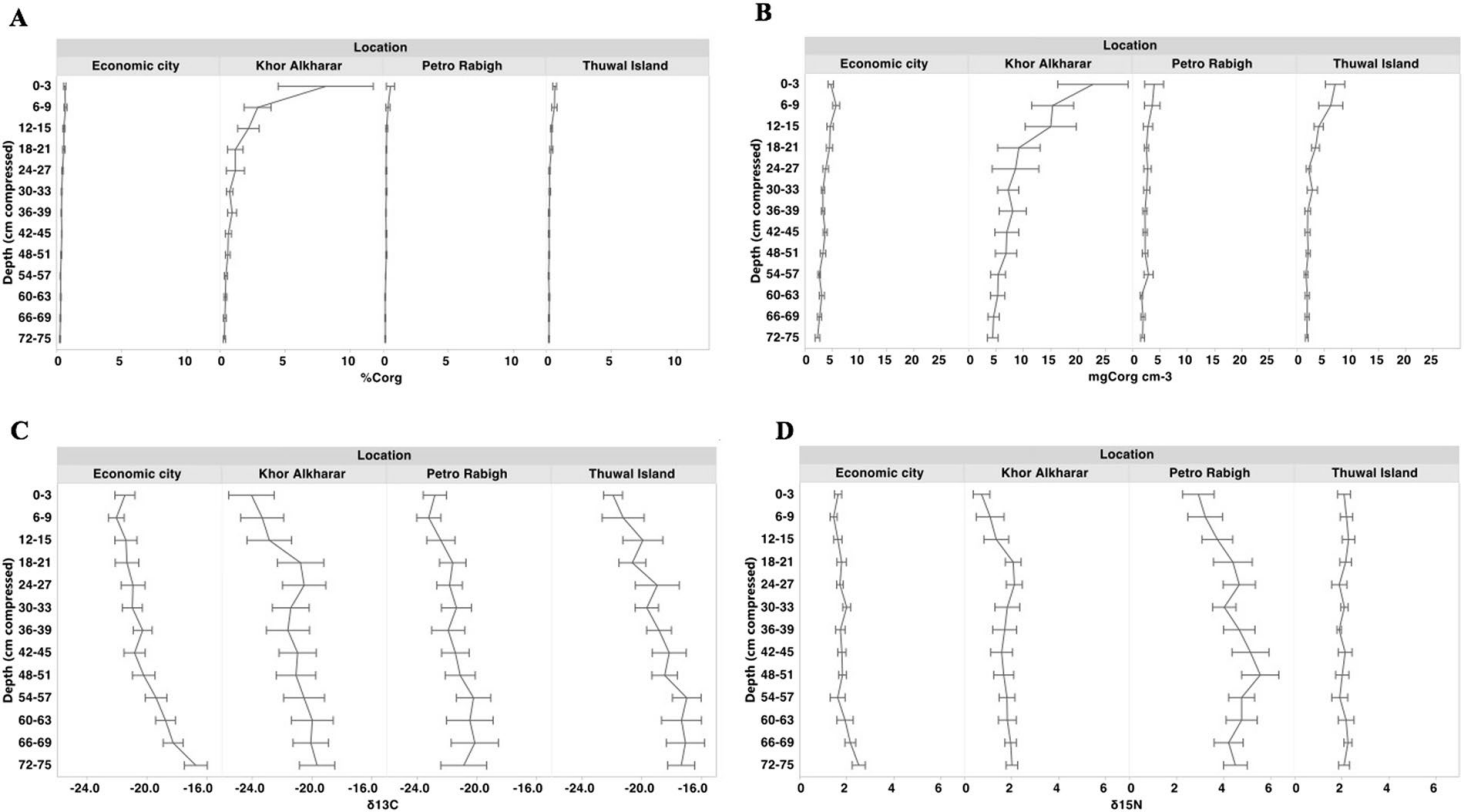
Vertical profiles of (**A**) % organic carbon (C_org_), (**B**) C_org_ density (g C_org_ cm^−3^), and (**C**,**D**) δ^13^C and δ^15^N (‰) in mangrove soils in central Red Sea. It was not possible to plot average values for the replicate cores against decompressed depths because replicate cores experienced different degrees of compaction during coring.

The C_org_ density and % C_org_ were generally low, ranging from 3 to 9 mg C_org_ cm^−3^ and 0.2% to 1.5% C_org_ (Table 2). The C_org_ density and % C_org_ significantly decreased with soil depth, while δ^15^N and δ^13^C significantly increased with depth, except for δ^15^N signatures at Thuwal Island which remained constant (Tukey HSD post hoc test, *P* < 0.05, Fig. 1 and Table 2). Mangrove soils in Khor Alkharar were characterized by relatively high soil C_org_ density (1 to 39 mg C_org_ cm^−3^ along the 170 cm soil profile), and high % C_org_ (0.2 to 17% C_org_ in the upper 25 cm), compared to the other locations (<14 mg C_org_ cm^−3^ and <1% C_org,_ Tukey HSD post hoc test, *P* < 0.05, Fig. 1). δ^13^C values did not differ among locations, whereas soil at Petro Rabigh mangroves had higher δ^15^N values (0.6 to 7.8‰ along the 170 cm soil profile) compared with those in other locations (<3.2‰ along the 170 cm soil profile) (Tukey HSD post hoc test, *P* < 0.05; Fig. 1 and Table 2).

**Table 2:** Mean (±SE) C_org_ density, % organic carbon (C_org_) and C and N stable isotope ratios of mangrove soil from four different locations in the Central Red Sea.

Location	*n* cores	Soil C_org_ Density (mg C_org_ cm^**−**3^)	% C_org_	Isotopes	
				δ^13^C	δ^15^N
Thuwal Island	65	3 ± 0.3^b^	0.3 ± 0.03^b^	−19 ± 0.3^a^	2 ± 0.1^b^
Economic City	104	4 ± 0.2^b^	0.4 ± 0.02^b^	−20 ± 0.2^a^	2 ± 0.1^b^
Petro Rabigh	78	3 ± 0.2^b^	0.2 ± 0.03^b^	−21 ± 0.3^a^	4 ± 0.2^a^
Khor Alkharar	65	9 ± 1^a^	1.5 ± 0.38^a^	−21 ± 0.4^a^	2 ± 0.1^b^
***R*** ^**2**^		0.8	0.8	0.8	0.8
***F*** **ratio** (location)		7.3^**^	5.8^**^	1.8 ^ns^	12.6^**^
***F*** **ratio** (depths nested-within-cores)		14^**^	15.7^**^	17.8^**^	^5^^**^
**All**	312	4 ± 0.3	0.6 ± 0.1	−21 ± 0.2	3 ± 0.1

*R* ^2^ and *F* ratio correspond to the square-root results of the GLMMs testing the effect of independence of samples. Study site and soil depth (nested within cores) as fixed factors, whereas replicate cores within sites was treated as random factor. *P value between 0.01 and 0.05, **P value < 0.01 for significant differences whereas (^ns^) means not significant. Columns linked with the same letter did not differ significantly among themselves (Tukey HSD multiple comparison post-hoc test, *P* > 0.05).

**Figure 2:**
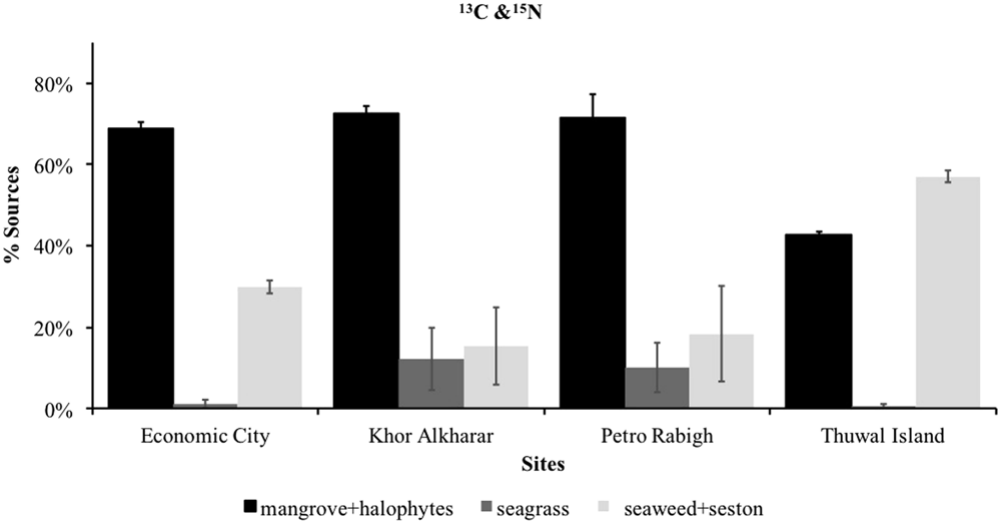
The sources of soil organic carbon (C_org_) in mangrove forests obtained using IsoSource from potential organic matter sources and soil δ^15^N and δ^13^C values.

The seaweed species analyzed (i.e. *Padina*, *Colpomenia*, *Turbinaria* and *Sargassum* species, Table 3) were unusually enriched in δ^13^C and, therefore, quite similar in carbon isotopic values to seagrass. This was the case also for seston, suggesting that it is mostly comprised of seagrass and macroalgal detritus in the mangrove waters sampled. Hence, the discrimination between these three sources (seaweed, seston and seagrass) was poor and involved considerable uncertainty (Table 3). Likewise, halophytes and mangroves had comparable δ^13^C signatures, but much lighter than those derived from plants performing aquatic photosynthesis (Table 3). The analysis of δ^15^N and δ^13^C values of marine plants and soil indicated that the source of the C_org_ in mangroves soil from the Red Sea was composed, on average, of 2/3 of C_org_ derived from atmospheric photosynthesis (e.g. mangrove and halophytes) and 1/3 of C_org_ derived from aquatic photosynthesis (e.g. seaweed, seston and seagrass, Tables 2 and 3, Fig. 2).

**Table 3:** Mean (±SE) of isotopic carbon and nitrogen values of marine plants (‰) collected at the four study sites. N indicated the number of samples analyzed.

Sources	Site	*n*	δ^13^C	δ^15^N
Mangrove and halophytes	Thuwal Island	39	−26 ± 0.2	2 ± 0.2
	Economic City	39	−27 ± 0.2	1 ± 0.3
	Petro Rabigh	24	−24 ± 0.9	5 ± 0.3
	Khor Alkharar	42	−25 ± 0.6	2 ± 0.4
Seagrass	Thuwal Island	6	−8 ± 0.4	−1 ± 0.3
	Economic City	39	−7 ± 0.2	−1 ± 0.2
	Petro Rabigh	27	−10 ± 0.3	2 ± 0.4
	Khor Alkharar	36	−9 ± 0.3	1 ± 0.2
Seaweed	Thuwal Island	27	−14 ± 0.7	2 ± 0.1
	Economic City	3	−8 ± 0.6	2 ± 0.2
	Petro Rabigh	—	—	—
	Khor Alkharar	18	−12 ± 0.8	2 ± 0.1
Seston	Thuwal Island	3	−14 ± 1.3	3 ± 0.1
	Economic City	3	−4 ± 2.1	2 ± 0.2
	Petro Rabigh	3	−17 ± 0.9	3 ± 0.1
	Khor Alkharar	3	−11 ± 2.5	2 ± 0.6

**Figure 3:**
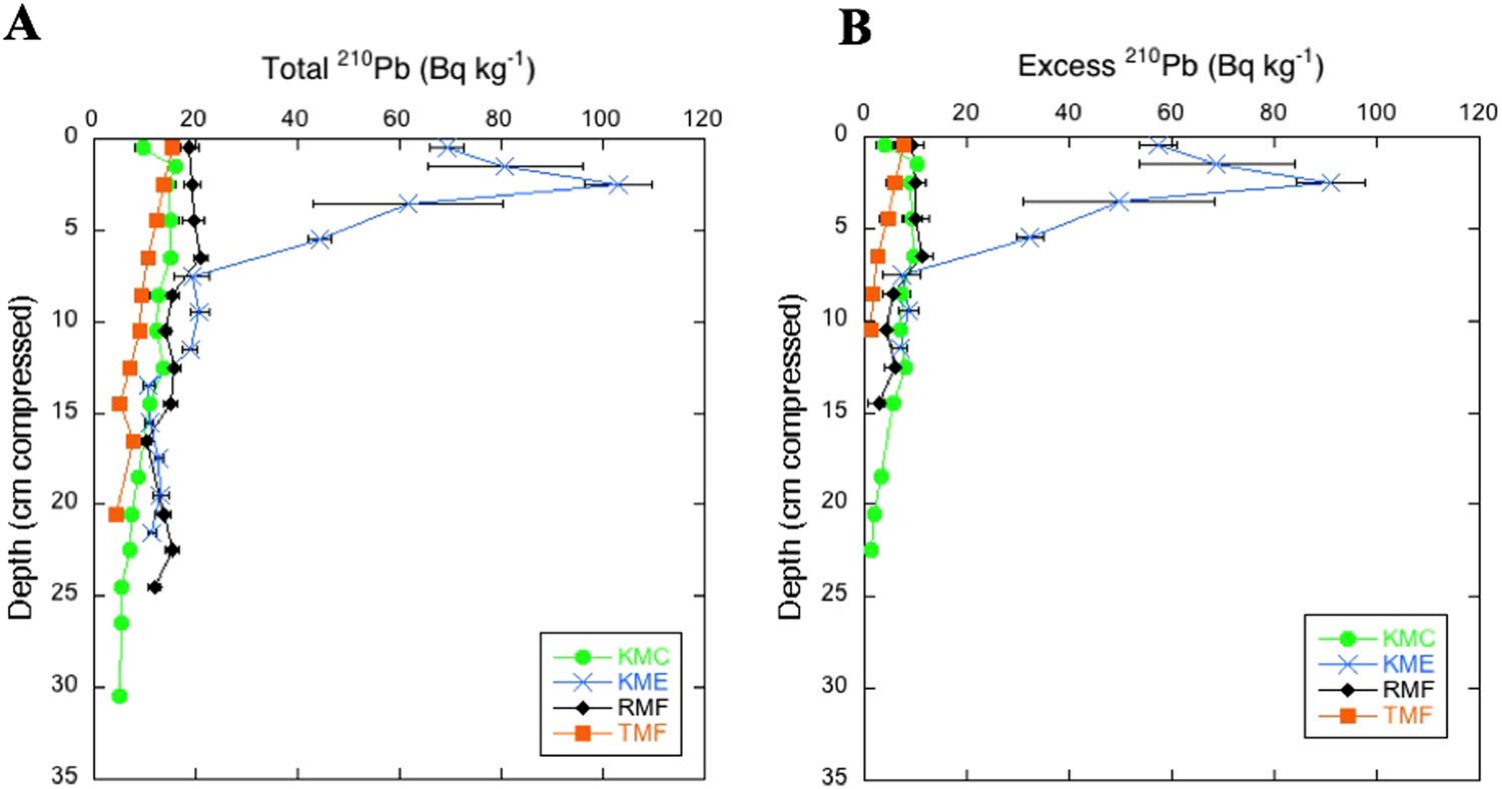
(**A**) Total and (**B**) excess concentration profiles of ^210^Pb in dated mangrove soil cores of Central Red Sea. It was not possible to plot average values for the replicate cores against decompressed depths because replicate cores experienced different degrees of compaction during coring.

The ^210^Pb concentration profiles of 6 out of 10 cores showed intense mixing of the upper layers or did not contain excess ^210^Pb, i.e. ^210^Pb concentrations were not significantly different than supported ^210^Pb and, thereby, it was not possible to apply a suitable ^210^Pb model to determine soil accretion rates (SAR). The ^210^Pb concentration profiles of 3 mangrove soil cores (KMC, KME and RMF) showed evidence of mixing in the upper 2 to 7 cm (Fig. 3a), and no apparent mixing in the top layers was observed for core TMF. Excess ^210^Pb concentrations at the surface were low, averaging 9 ± 2 Bq kg^−1^ in all cores, except for KME, which reached 90 Bq kg^−1^. Excess ^210^Pb concentrations decreased steadily below the surface mixed layer (when present) and down to 22, 12, 15 and 11 cm, in cores KMC, KME, RMF and TMF respectively. Supported ^226^Ra concentrations were similar among the 10 analyzed cores, averaging 10 ± 3 Bq kg^−1^, with KMC concentrations being significantly lower at 5.6 ± 0.6 Bq kg^−1^ (Fig. 3b, Table S5). The CF:CS model (Constant Flux: Constant Sedimentation rate^24, 25^) was applied to these cores (below the mixed layer when present), obtaining SAR ranging from 0.7 ± 0.1 to 3.7 ± 0.7 mm yr^−1^ over the last 100 years. Due to the presence of mixing, these rates must be considered as upper limits, especially for KMC and RMF. The resulting SAR averaged 2.2 ± 0.6 mm yr^−1^ over the past 100 years (Table 4). The range of estimates of SAR over longer time-scales derived from ^14^C age estimates were comparable (0.4 to 1.8 mm yr^−1^, Table 5) to those derived for the past century, while the average was 2.6 times lower. Long-term SAR did not differ among locations (Table 5, Tukey HSD post hoc test, *P* > 0.05).

**Table 4:** Mean (±SE) organic carbon (C_org_) stocks in 10 cm-thick soils, and short-term (since 1900) soil accretion rates and soil C_org_ sequestration rates based on ^210^Pb from four different locations in the Central Red Sea.

Location	*n* cores	C_org_ Stock-in 10 cm thick soil	^210^Pb soil accretion rate	Carbon sequestration using ^210^Pb (normalized to 1900)
		g C_org_ m^−2^	mm yr^−1^	g C_org_ m^−2^ yr^−1^
Thuwal Island	8	530 ± 146^b^	2.1 ± 0.7	10 ± 3^c^
Economic City	8	531 ± 58^b^	2.2 ± 0.7^*^	16 ± 6^b^
Petro Rabigh	6	382 ± 153^b^	2.4 ± 1.0	9 ± 4^d^
Khor Alkharar	7	1541 ± 430^a^	0.7 ± 0.1 to 3.7 ± 0.7	23 ± 1^a^
***R*** ^**2**^		0.4	0.1	0.9
***F*** **ratio**		5^**^	0.8 ^ns^	10030^**^
**All**	29	744 ± 140	2.2 ± 0.7	15 ± 1

*R* ^2^ and *F* ratio correspond to an ANOVA testing for significant differences between locations. *P value between 0.01 and 0.05, **P value < 0.01 for significant differences between depths, whereas (^ns^) means not significant. Columns linked with the same letter did not differ significantly among themselves (Tukey HSD multiple comparison post-hoc test, *P* > 0.05).

*Here refers to the mean sediment accretion rate of the locations where ^210^Pb could be used to establish a geochronology.

**Table 5:** Mean (±SE) organic carbon (C_org_) stocks in 1 m-thick soils, and long-term (millennia) soil accretion rates and soil C_org_ sequestration rates based on ^14^C from four different locations in the Central Red Sea.

Location	*n* cores	C_org_ Stock-in 1 m thick soil	^14^C soil accretion rate	Carbon Sequestration using ^14^C
		g C_org_ m^-2^	mm yr^-1^	g C_org_ m^-2^ yr^-1^
Thuwal Island	8	3034 ± 416^b^	1.8 ± 1^a^	5.5 ± 3.4^a^
Economic City	8	3838 ± 291^b^	0.4 ± 0.1^a^	1.6 ± 0.2^a^
Petro Rabigh	6	2471 ± 470^b^	0.6 ± 0.1^a^	1.5 ± 0.4^a^
Khor Alkharar	7	7618 ± 1530^a^	0.6 ± 0.2^a^	5 ± 2.8^a^
*R* ^2^		0.5	0.1	0.1
*F* ratio		7.8^**^	1.2 ^ns^	0.8 ^ns^
All	29	4246 ± 533	0.9 ± 0.3	3.5 ± 1.1

*R* ^2^ and *F* ratio correspond to an ANOVA testing for significant differences between locations. *P value between 0.01 and 0.05, **P value < 0.01 for significant differences between depths, whereas (^ns^) means not significant. Columns linked with the same letter did not differ significantly among themselves (Tukey HSD multiple comparison post-hoc test, *P* > 0.05).

The high C_org_ content at Khor Alkharar resulted in the highest C_org_ stock at this site, which was significantly higher compared to all other locations (Tables 4 and 5). Likewise, recent C_org_ sequestration rates averaged 15 ± 1 g C_org_ m^−2^ yr^−1^ (i.e. based on ^210^Pb dating) and varied three-fold, from the lowest rates at Petro Rabigh (9 ± 4 g C_org_ m^−2^ yr^−1^) to the highest rates at Khor Alkarar (23 ± 1 g C_org_ m^−2^ yr^−1^; Table 4, Tukey HSD post hoc test, *P* < 0.05). These differences were driven mostly by the differences in C_org_ density, as soil accretion rates were rather uniform and did not differ significantly among locations. The long-term C_org_ sequestration rates did not differ among locations, averaging 3.5 ± 1.1 g C_org_ m^−2^ yr^−1^ (Table 5, Tukey HSD post hoc test, *P* > 0.05).

## Discussion

The sediment grain size we found was consistent with the findings of Gheith & Abou-ouf^26^ who reported sandy sediments in Khor Alkharar Sea becoming coarser, with a higher gravel component, towards the beach, and sediment at Rabigh to be mainly composed of medium grain size sand. Although, the clay and silt content of mangrove soils at Khor Alkharar were relatively low compared to the other locations, site KME had considerably high content of mud 24% (average top 20 cm; soil thickness where excess ^210^Pb is found), hence the high content of fine sediments at surface layers together with high concentrations of C_org_ can explain the higher inventory of excess ^210^Pb found at this site compared to other locations.

The average (±SE) C_org_ density in Central Red Sea mangrove soils (0.0044 ± 0.00028 g C_org_ cm^−3^) is remarkably low compared to values reported from a global compilation (e.g. average 0.055 g C_org_ cm^−3^ and minimum 0.023 g cm^−3^ in Kosrae Island ^27^, or 0.038 g C_org_ cm^−3^ and 0.061 g C_org_ cm^−3^ characteristic of estuarine and oceanic mangrove soils, respectively^14^). The C_org_ density in the mangrove soils of the Central Red Sea is 100 to 300-fold lower than in mangrove soils in wet temperate to subtropical climate at Rookery Bay, Florida (0.51 g cm^−3^; ref. 28) and dry tropical climate at Abu Dhabi, UAE (1.2 g cm^−3^; ref. 29). Soil C_org_ stocks are highly variable across hemispheres, latitudes, countries and plant community compositions^30^, thereby the relatively lower C_org_ density values found at Central Red Sea could be explained by particular habitat characteristics and geomorphological settings not favorable for C_org_ sequestration.

In contrast, the recent soil accretion rates of Central Red Sea mangroves soil (mean 2 mm yr^−1^) are close to the median global value of 2.8 mm yr^−1^ ^29^ and half of the median value of 4.5 mm yr^−1^ reported for mangroves globally^3^, and within the range previously reported (0.1 to 21 mm yr^−1^ ^3, 8^). Moreover, the values obtained for the Red Sea mangroves using ^210^Pb chronologies are higher than those recently reported using comparable methods for mangroves in Moreton Bay and southeastern Australia (1.2 and 1.7 mm yr^−1^, respectively^31^), and lower than the average rate reported in Florida (2.7 mm y^−1^)^32^.

The SAR obtained using the ^210^Pb method (for short-term periods, last 100 years) is up to 4-fold higher than those based on ^14^C (1 mm yr^−1^), which encompass long-term (millennia) accretion, in areas such as Economic City, Petro Rabigh and Khor Alkharar. These results suggest an increase in SAR during the last decades, which is consistent with coastal development in the Saudi coast of the Central Red Sea, that experienced industrial and urban developments over the past decades. This is supported by the high value of ^15^N, that suggests an increase of nutrients from land runoff and coastal development as sewage and fertilizers are released to the sea. Moreover, decreasing δ^13^C values and increasing mud content (silt and clay) towards recent periods also support this hypothesis. However, the presence of mixing in top layers, decomposition of organic matter with ageing, compaction of soils during diagenesis, and intrinsic differences in ^210^Pb and ^14^C methodologies could also explain the higher SAR found in recent times^33–35^.

Whereas SAR are not particularly low for Central Red Sea mangrove forests, their remarkably low soil C_org_ density resulted in low carbon sequestration rates. The average soil C_org_ sequestration rates of 15 g C_org_ m^−2^ yr^−1^ for Central Red Sea mangroves soils is 10-fold lower than the average value of 163 g C_org_ m^−2^ yr^−1^ reported for mangroves globally^29^, and are in the low range of values previously reported (10 to 920 g C m^−2^ yr^−1^)^8^. The long-term (millenary time scale) soil C_org_ sequestration rates in Central Red Sea mangroves (3.5 g C_org_ m^−2^ yr^−1^) was also well below the soil C_org_ sequestration rates derived from ^14^C chronologies reported for dwarf *A*. *germinans* forests in arid Baja California (256 g C_org_ m^−2^ yr^−1^)^23^ and Pohnpei Island, Micronesia dominated by *Rhizophora apiculata*, (93 g C_org_ m^−2^ yr^−1^)^29^, despite SARs at the Micronesia sites being comparable to those reported here for Red Sea mangroves, of 2 mm yr^−1^ ^36^. Despite of the low long-term sequestration of carbon in Central Red Sea mangroves compared to other mangrove habitats, their capacity to sequester C_org_ is similar to that observed in tropical forest soils (2.3 to 2.5 g C_org_ m^−2^ yr^−1^)^37^.

The relatively low C_org_ stocks and C_org_ sequestration rates in Red Sea mangroves are most likely due to the oligotrophic nature and low allochthonous inputs to the Red Sea. The lack of rivers and the extremely arid conditions result in nutrient-limited mangrove growth^19^, reflected in low-biomass dwarfed trees, particularly within the study region in the Central Red Sea^20^. Moreover, the soils of Red Sea mangroves are mainly composed of biogenic coarse carbonates, which could also explain the relatively low C_org_ sequestration capacity of mangroves growing in unfavorable conditions for biomass production, soil accretion and preservation compared to mangrove habitats from temperate and sub-tropical habitats. The isotopic results showed that two-thirds of the soil C_org_ stocks originated from mangrove or tidal-marsh biomass, while one-third was derived from marine photosynthetic organisms. Previous studies demonstrated that terrigenous C_org_ inputs can contribute up to 30% of C_org_ stocks in mangrove soil associated with riverine ecosystems^38^, but the lack of rivers implies that there is no influx of riverine soil and organic matter in coastal areas and, therefore, C_org_ sequestration in Red Sea mangroves is limited to autochthonous production and fluxes from the ocean.

The lack of terrigenous inputs is also reflected in the heavy carbon isotopic signatures of suspended particulate matter (−11.3 ±1.6‰), indicative of sestonic organic matter of marine origin, as seston from river sources is characterized by lighter values ranging from −18.5 to −26.4‰^39^. Moreover, the seston carbon seems to be dominated by carbon derived from macrophytes as plankton-derived C_org_ is also relatively depleted in ^13^C (−24.7 to −26.0‰ in the open ocean, and −19.8 to −22.3‰ in semi-tropical regions)^40^.

We hypothesized that in contrast with other mangrove forests, CO_2_ capture by mangroves in the Red Sea would be extremely low due to the arid conditions of this region. Indeed, poor C_org_ preservation under coarse grained-soils, high hydraulic conductivity, and low moisture-holding capacity, could also result in low C_org_ storage, as previously demonstrated for mangroves from United Arab Emirates^41^. Considering the total mangrove area in the Red Sea (135 km^2^)^18^, the rates reported for Red Sea mangroves here represent about 2100 tons of carbon sequestered per year, which is a relatively low contribution. Whereas Red Sea mangroves remains a healthy and stable ecosystem in the Red Sea^18^, they offer a limited potential to support blue carbon strategies to mitigate CO_2_ emissions.

## Methods

### Study location, sampling and laboratory procedures

**Figure 4:**
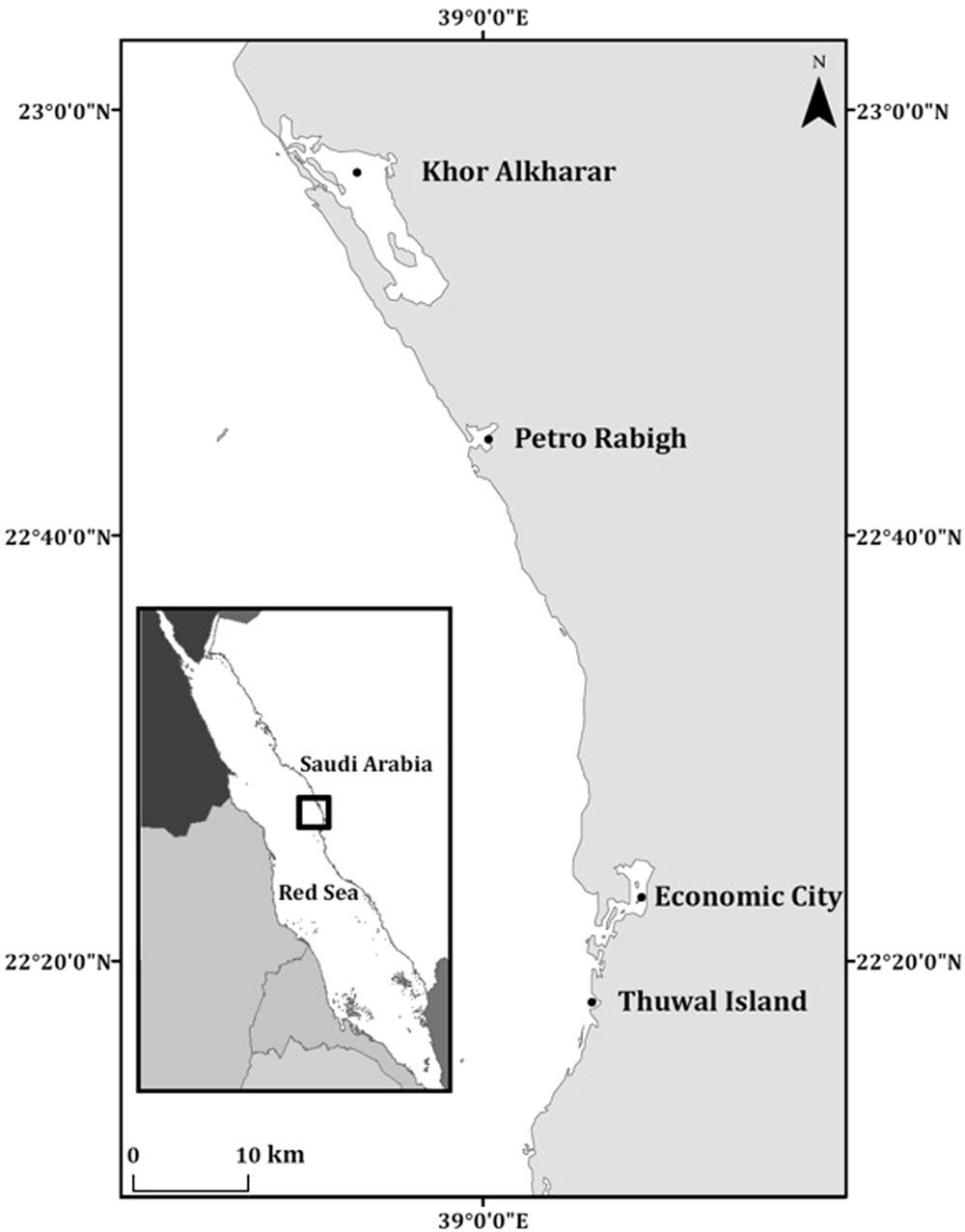
Location of the Central Red Sea mangrove forests sampled. The map was produced with ArcMap Version 10.2. Background map credits: the World Administrative Divisions layer provided by Esri Data and Maps, and DeLorme Publishing Company. Redistribution rights are granted http://www.esri.com/~/media/Files/Pdfs/legal/pdfs/redist_rights_103.pdf?la=en.

Sampling was conducted in mangrove forests of *Avicennia marina* at Thuwal Island, Economic City, Petro Rabigh and Khor Alkharar (Kingdom of Saudi Arabia, Fig. 4). The area encompassed by this study extends along 80 km of coastline, from Thuwal Island where mangroves grow on a shallow soil of weathered coral^42^, to Khor Alkharar, a coastal lagoon permanently connected to the Red Sea^43^. Petro Rabigh is a major industrial and petrochemical complex, whereas Economic City, about 40 km south of Petro Rabigh, is a newly developed city and harbor complex^26, 44^. Thuwal Island and Khor Alkharar lagoon are relatively away from direct sources of human disturbance whereas mangrove forests near Petro Rabigh and the Economic City are subjected to disturbances caused by industrial and coastal development, respectively.

In order to assess the C_org_ sink capacity of Red Sea mangroves, a total of 29 soil cores were sampled: 8 cores at Thuwal Island, 8 cores at Economic City, 6 cores at Petro Rabigh and 7 cores at Khor Alkharar (Fig. 4 and Table S1 in Supplementary materials). The soils were sampled using manual percussion and rotation of PVC pipes (170 cm long, high pressure class 12 with an inner diameter of 62.6 mm) that were gently hammered into the soil (<0.5 m water depth). The top core was sealed with PVC tape before retrieval to create vacuum and avoid losing the sample during recovery. The cores were sealed at both ends and transported vertically to the laboratory for further processing. Half of the cores collected at each location were kept intact and transported to the laboratory (hereafter referred to as ‘whole cores’). The other cores from each study location were sampled using a corer consisting of a PVC pipe with pre-drilled holes in the sidewall (3 cm wide and 3 cm apart; hereafter referred to as ‘port cores’), allowing sub-sampling of soil samples along the core in the field by inserting 60 ml syringes into the pre-drilled holes along the PVC pipes. The length of the core barrel inserted into the soil and the length of retrieved mangrove soil were recorded in order to correct the core lengths for compression effects and all variables studied here are referenced to the corrected, uncompressed depths. The volume of each subsample retrieved from the port cores was recorded in the field. The whole cores were sealed at both ends and transported vertically to the laboratory together with the subsamples obtained from the port cores.

PVC whole cores were cut lengthwise and cut into 1 cm-thick slices. Each slice from the whole cores and the subsamples from the port cores were weighed before and after oven drying at 60 °C until constant weight (dry weight; DW) to estimate dry bulk density (DBD in g DW cm^−3^). Then, every second slice of whole cores and all subsamples from port cores were divided into two subsamples by quartering. One subsample was ground and analyzed for organic carbon (C_org_), and stable carbon and nitrogen isotopic composition (δ^13^C and δ^15^N), and the other subsamples were used for soil grain-size, ^14^C and ^210^Pb analyses.

Samples were acid-rinsed to ensure complete removal of inorganic carbon (i.e., carbonates) before C_org_ analysis, despite the fact that this procedure may lead to an underestimation of soil C_org_ stocks^45, 46^. For C_org_, δ^13^C and δ^15^N analyses, 1 g of ground samples was acidified with 1 M HCl until bubbling stopped to remove inorganic carbon, centrifuged (3500 RPM; 5 min) and the supernatant with acid residues was removed using a pipette, then washed in deionized water once, the residues were centrifuged again and the supernatant removed. The residual samples were re-dried (70 °C) and encapsulated for analysis using a Thermo Delta V Conflo III coupled to a Costech 4010 at the UH Hilo Analytical Laboratory, USA (Table S2 in Supplementary materials). The content of C_org_ was calculated for the bulk (pre-acidified) samples. Organic carbon and Nitrogen isotope ratios are expressed as δ values in parts per thousand and relative to the Vienna Pee Dee Belemnite and atmospheric nitrogen using USGS 40 and USGS 41 standards, respectively. Analyses of replicates and reference materials were carried out to ensure reproducibility of the results.

The carbon and nitrogen elemental and isotopic composition was also analyzed in *Avicennia marina* macro-detritus (i.e. aerial roots, green and senescent leaves, stem, buds, flowers), halophytes (e.g. *Salicornia* spp. etc.), seaweeds (i.e. *Padina*, *Colpomenia*, *Turbinaria* and *Sargassum* spp.), seagrasses (i.e. rhizomes, roots and leaves from *Halophila*, *Halodule*, *Thalassodendrum*, *Thalassia* and *Enhalus* spp.) and seston particulate organic matter (fraction retained on a 0.7 μm pore diameter filter) collected at the four study locations. The samples from living materials were milled, and encapsulated for elemental and isotopic analyses as described above. Living material containing carbonates in their tissues were acidified with 1 M HCl before analyses (see Table S3 in Supplementary materials). The seston filters were pretreated with acid using the fumigation method^47^. In total 312 samples for carbon and nitrogen isotopic composition of putative carbon sources were analyzed.

For soil grain-size analysis, a Mastersizer 2000-Malvern was used following sieving (1 mm) digestion of <1 mm samples with 30% hydrogen peroxide at the Centro de Estudios Avanzados de Blanes, Spain. Grain size classification and texture were categorized following the Wentworth scale^48^.

The concentrations of ^210^Pb in the upper 20 to 30 cm of one to two cores per location were determined in the soil fraction <125 μm at the Universitat Autònoma de Barcelona (Spain) through the measurement of its granddaughter ^210^Po assuming radioactive equilibrium between both radionuclides^49^. Briefly, after addition of a known amount of ^209^Po as yield tracer, samples were acid digested in an analytical microwave, the polonium isotopes were auto-plated in silver discs and the concentration of ^210^Po quantified by alpha spectrometry. The concentrations of excess ^210^Pb used to obtain the age models were determined as the difference between total ^210^Pb and ^226^Ra (supported ^210^Pb). Concentrations of ^226^Ra were determined for selected samples along each core by low-background liquid scintillation counting method (Wallac 1220 Quantulus)^50^. These concentrations were found to be in agreement with the concentrations of total ^210^Pb at depth below the excess ^210^Pb horizons. Analyses of reagent blanks, replicates and a reference material (IAEA - 315, marine sediment) were carried out for both ^210^Pb and ^226^Ra to assess for any contamination and to ensure reproducibility of the results, (Table S4 in Supplementary materials).

A total of 94 radiocarbon analyses were conducted in 25 of the 29 cores sampled (2–5 cores per location) at two soil depths per core (cm 18–21 and the bottom cm towards the end for the ‘port core’, and cm 40 and the bottom cm towards the end for the ‘whole core’), at the AMS Direct Laboratory (USA). Samples consisted of pooled shells and bulk soil, (Table S5 in Supplementary materials).

The IsoSource software package^51^ was used, using δ^13^C and δ^15^N, to estimate the proportion of the C_org_ in the soil derived from different plants with atmospheric (e.g. *A*. *marina* and halophytes) and aquatic (e.g. seaweeds, seagrasses and suspended particulate organic matter ‘seston’) photosynthesis collected at the four study sites, using a 1% increment and 0.1 to 0.5‰ tolerance.

The soil C_org_ stocks per unit area (g C_org_ m^−2^) were estimated for 10 cm and 1 m soil thicknesses (i.e. cumulative mass). Where necessary (i.e. in 7 cores), we inferred C_org_ stocks below the limits of the reported data to 1 m, by extrapolating linearly the cumulative C_org_ stocks to 1 m.

Soil C_org_ sequestration rates (expressed in g DW m^−2^ y^−1^) for the last century and the last millennia were estimated using ^210^Pb (CF:CS model)^24^, and ^14^C age models, respectively. Mean soil accretion rates (SAR; mm y^−1^) over the last 200 to 5000 years (i.e. based on ^14^C) were determined by calibrating the raw radiocarbon dates reported by the Laboratory using the R routine “Bacon” (Marine13 curve) for Bayesian chronology building^52^ and corrected for the marine reservoir effect (i.e. subtracting Delta R value of 110 ± 38 for the Red Sea)^53^. From the Bacon routine output, the mean age was used to produce an age-depth weighted regression model forced through 0 (0 cm is cal. BP: 1950), using as weight the sum of the Euclidean distance of the minimum and maximum ages.

Sequestration rates of C_org_ were estimated by dividing the inventories in 100 cm-thick soil by the average soil accretion rate derived from ^14^C, whereas, the ^210^Pb-derived sequestration rate was calculated for 10 cm-thick soils by multiplying the soil accretion rate by the fraction of C_org_ accreted since 1900. For the cores that were not possibly dated, we assumed they supported the same accretion rates as the dated cores at the same location. The cores from Economic city could not be successfully dated with ^210^Pb (i. e. showed mixing) and we assumed that the soil accretion rate of that area is the average of the successfully dated cores in other locations.

Statistical analyses were carried out using JMP software, including descriptive statistics and ANOVA (for all 29 cores), and General Linear Mixed Models (GLMM) (for 25 cores, as cores less than 1 m long were excluded) to test for differences among forests, followed by Tukey HSD posthoc tests to assess pairwise differences among sites and soil depths.

Generalized Linear Mixed Models (GLMM) were used to take into account the potential non-independence of samples taken at different depths within the same core, since depth is a proxy for time in the cores. And given the spatial separation of cores within mangrove forests (hundreds of meters) we considered the cores themselves to be spatially independent. All response variables (bulk density, C_org_, δ^13^C signatures and soil grain size fractions) were square-root transformed prior to analyses to homogenize their variances. Study sites (Thuwal Island, Economic City, Petro Rabigh and Khor Alkharar) and soil depth nested within cores were treated as fixed factors, whereas replicate cores within sites was treated as random factor.

## Electronic supplementary material

Supplementary Materials
